# Personalized tutoring narrows the clinician-faculty gap in *General Surgery* exam scores among vocational medical students

**DOI:** 10.1186/s12909-025-08371-5

**Published:** 2025-12-01

**Authors:** Jianya Cai, Wenting Li, Shuangta Xu

**Affiliations:** 1 Department of Surgery, Quanzhou Medical College, Quanzhou, 362000 China; 2 Thyroid and Breast Surgery Department, Quanzhou Maternity and Children’s Hospital, Quanzhou, Fujian China; 3 Department of Surgery, The Second Affiliated Hospital of Fujian Medical University, Quanzhou, China; 4 Department of Surgery, The Second Clinical Medical College of Fujian Medical University, Quanzhou, 362000 China

**Keywords:** Teaching methods, Vocational medical students, General surgery, Exam scores, Personalized tutoring

## Abstract

**Introduction:**

To explore the impact of teaching by clinical educators and faculty educators on the academic performance of vocational medical students, this study analyzed the final exam scores of four classes in the *General Surgery* course (2023 cohort) at our institution’s School of Clinical Medicine.

**Methods:**

This study conducted a questionnaire survey of 154 students in the 2023 cohort of the clinical medicine major at our college and collected their final exam scores for the *General Surgery* course. All data were statistically analyzed using R software and GraphPad Prism 9.5.1.

**Results:**

Clinical educators are more effective in integrating theoretical knowledge with clinical practice, but they show deficiencies in classroom discipline, learning supervision, and teacher-student interaction. Personalized tutoring can significantly improve the academic performance of students in clinician-taught class, bringing their grades to a level comparable to those in courses taught by faculty educators.

**Conclusion:**

This study demonstrates that personalized tutoring effectively improves written exam performance in clinician-taught classes to levels comparable with faculty-taught classes, while faculty-taught classes show no significant benefit from additional tutoring. We recommend targeted pedagogical training for clinical educators and implementation of a clinician-faculty collaborative teaching model to better serve vocational medical students’ learning needs.

**Supplementary Information:**

The online version contains supplementary material available at 10.1186/s12909-025-08371-5.

## Introduction

 The Chinese medical education system features tiered academic programs, comprising: the three-year vocational diploma, five-year undergraduate degree, ‘5 + 3’ integrated master’s pathway, and eight-year direct doctoral program [1, 2]. Among these, the 3-year vocational medical education plays a critical role in cultivating healthcare professionals for primary care, particularly by providing a significant number of medical personnel for rural and underdeveloped regions [3, 4]. In the first year, vocational medical students primarily focus on basic medical courses; in the second year, they begin clinical courses; and in the third year, they engage in medical internships [1]. Compared with medical students from other academic tracks, those in the three-year vocational program typically achieved lower Gaokao scores (China’s national college entrance exam). This likely reflects their relatively weaker foundational knowledge, learning aptitude, and self-regulation skills [5]. Additionally, the 3-year curriculum requires them to acquire extensive medical knowledge in a shorter time frame, thereby increasing their academic pressure. These factors create unique learning needs for vocational medical students, who require more targeted educational methods, increased guidance, and closer attention from instructors [6]. In this context, the teaching methods and instructional models adopted by educators become critical. Therefore, understanding the impact of different types of instructors and their teaching styles on the academic achievement of vocational medical students is of great significance for optimizing educational practices and enhancing the quality of medical education for this group.

Currently, clinical courses for vocational medical students are typically taught by two types of instructors: In one group, the course was team-taught by clinical educators, with each instructor covering material relevant to their specialty area. The comparison group involved full-time college instructors, all possessing relevant teaching credentials, with individual instructors assuming complete responsibility for course delivery. The advantage of multiple clinical educators teaching together is their ability to bring diverse clinical experiences, which helps students develop critical thinking and the ability to adapt to clinical problems flexibly [7, 8]. This is also the teaching method adopted by many undergraduate medical schools. However, this model may present challenges for students with weaker academic foundations. Variations in teaching pace and style among different clinical educators may make it difficult for students to adapt to the course material due to frequent teacher changes. Due to the heavy clinical workload and research responsibilities of clinical educators, they may not be able to dedicate sufficient time and effort to course preparation and student mentoring, which can negatively impact teaching quality [9]. Additionally, with multiple instructors, students may struggle to find a consistent mentor for feedback and discussion, which can delay the resolution of learning issues [10]. In contrast, full-time instructors provide a consistent teaching style, pace, and focus, allowing students to learn within a coherent framework and avoid confusion caused by teacher differences. Moreover, full-time instructors are better able to understand each student’s learning progress and offer more targeted guidance and support [11]. They can address student issues in a timely manner, improving teaching efficiency and quality. Students are also more likely to establish a trusting relationship with full-time instructors, facilitating deeper communication and individual mentoring [12, 13]. However, the full-time teaching model also has potential drawbacks. Firstly, due to the limited knowledge and experience of a single instructor, the course content may lack diversity and depth [14], in contrast to the model involving multiple clinical educators, which can offer a rich variety of clinical cases and multifaceted analyses. Secondly, full-time instructors may experience burnout from teaching the same course for an extended period, which could affect their enthusiasm for teaching and innovation [15, 16]. Additionally, if the instructor lacks sufficient clinical experience, students may face limitations in developing practical skills and clinical reasoning.

Building on this background, this study examines the final examination performance of four cohorts of 2023 Clinical Medicine students in their *General Surgery* course. The research aims to assess how different instructional approaches affect academic outcomes. These findings will provide empirical evidence to inform pedagogical improvements in vocational medical education and support student achievement.

## Methods

### Participants

The participants in this study were second-year Clinical Medicine students from Quanzhou Medical College, totaling 154 students. The study was conducted from September 2024 to April 2025. The study used pre-assigned classes (Class 1, 2, 3, and 4 of the 2023 cohort of Clinical Medicine students) as the study subjects, rather than random sampling or assignment, thus employing a quasi-experimental design.

### The inclusion criteria were as follows

(1) Second-year Clinical Medicine students from the 2023 cohort; (2) Students who were willing to participate in the various teaching activities, assessments, and surveys of the study; (3) Students who were able to attend the course regularly and participate in the final exam.

### The exclusion criteria were as follows

(1) Students who had received other educational interventions (such as off-campus tutoring, specific training courses, etc.); (2) Students who were unable to complete most of the course content due to absenteeism or other reasons; (3) Students who were unable to participate in the final assessment or the course satisfaction survey as required; (4) Students who had been retained from previous years and joined this grade.

### Experimental design

**Fig. 1:**
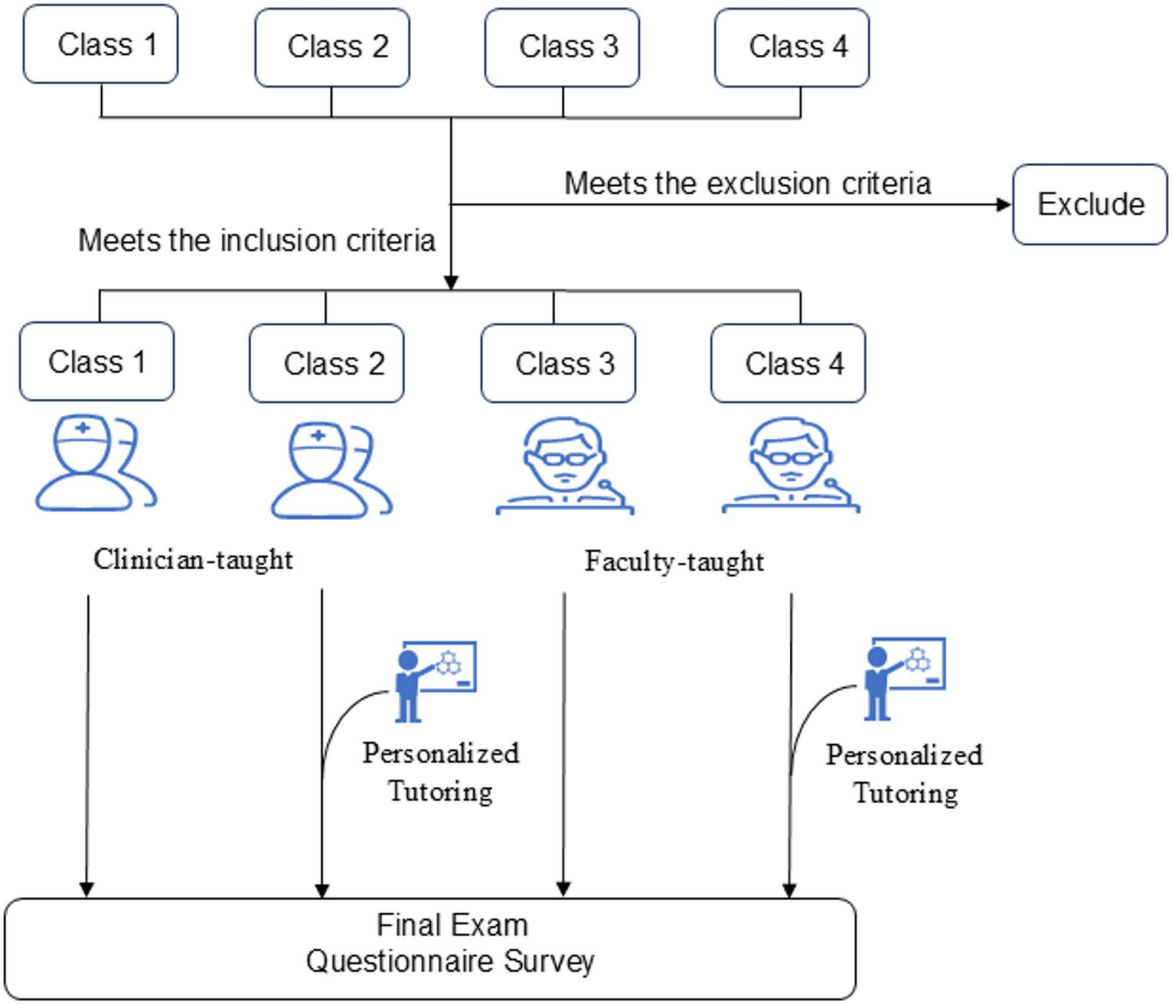
Experimental design flowchart

All participants followed the standard “*General Surgery*” curriculum, consisting of four weekly sessions over a 16-week period. While the syllabus content and instructional pacing were uniform across groups, variations existed in: (1) the type of instructor (clinical educators vs. faculty educators), and (2) provision of supplemental tutoring. The study period concluded with a comprehensive final examination and student satisfaction survey (Fig. 1).

### Teaching model

Class 1: Clinician-taught. This class consists of 32 students and is taught by 12 clinical educators from the Department of Surgery at the hospital. Each clinical educator is responsible for teaching between two and eight class hours.

Class 2: Clinician-taught with Personalized Tutoring. This class consists of 40 students and is taught by 12 clinical educators from the Department of Surgery at the hospital. Each clinical educator is responsible for teaching between two and eight class hours. A total of four personalized tutoring sessions were conducted throughout the semester. These sessions were delivered by a dedicated tutor, a full-time surgical faculty educator with substantial expertise in the General Surgery curriculum, who was not involved in teaching Classes 1–4. The tutor’s responsibilities included monitoring student progress and addressing student inquiries.

Class 3: Faculty-taught. This class consists of 44 students and is taught by a faculty educator from the college. The course instructor is responsible for delivering the entire course content, and no personalized tutoring sessions were arranged.

Class 4: Faculty-taught and Personalized Tutoring. This class consists of 38 students and is taught by a faculty educator from the college, who is responsible for delivering the entire course content. Additionally, four personalized tutoring sessions were provided by a dedicated tutor. This tutor was a full-time surgical faculty educator who did not teach Classes 1–4, with the same responsibilities as those described for Class 2, which included monitoring student progress and resolving learning difficulties.

### Final exam format and content

#### Exam format

The final examination was administered as a proctored, closed-book written assessment.

#### Exam content

The final exam covers all course material from the semester, with a focus on evaluating students’ understanding of basic surgical knowledge. The questions are drawn from the core topics of each unit in the course syllabus.

The final examination comprised two question types with distinct assessment objectives. Single-choice questions (70% weighted) evaluated students’ grasp of fundamental surgical concepts and clinical decision-making skills through items of varying difficulty levels, testing both factual knowledge retention and applied clinical reasoning abilities. The question-and-answer Sect. (30% weighted) assessed higher-order competencies including synthesis of core surgical knowledge and analytical proficiency in clinical case scenarios, requiring demonstration of integrated problem-solving skills.

### Scoring criteria

The final exam was graded according to a standardized scoring system. All papers were graded by professional surgical instructors to ensure objectivity and consistency. Single-choice questions were graded based on the standard answers, while question-and-answer sections were scored based on the completeness of key points in the answers.

### Questionnaire survey

This study designed a questionnaire survey to understand the teaching situations of different types of instructors. The question options are scored on a Likert five-point scale (1 = Very Negative, 2 = Negative, 3 = Neutral, 4 = Positive, 5 = Very Positive). The survey questions covered the following ten aspects:

1. Whether the lecture content aligns with key exam topics
2.  Clarity of knowledge point explanations
3. Emphasis on key and difficult concepts
4. Vividness and visual appeal of teaching materials
5. Integration of theory with clinical practice
6. Strictness of classroom discipline requirements
7. Instructor’s attention to students’ knowledge mastery
8. Teacher-student interaction
9. Instructor’s efforts to enhance student learning motivation
10.  Frequency of reviewing past knowledge points

### Statistical methods

Descriptive statistics characterized score distributions, with mean and standard deviation representing central tendency and variability respectively. Intergroup comparisons were conducted using: (a) independent samples t-tests for parametric comparisons between classes, and (b) Mann-Whitney U tests for non-parametric analyses. Logistic regression modeled associations between outcomes and predictor variables, estimating effect magnitudes for each covariate.

## Results

### Distribution of final exam scores

**Fig. 2:**
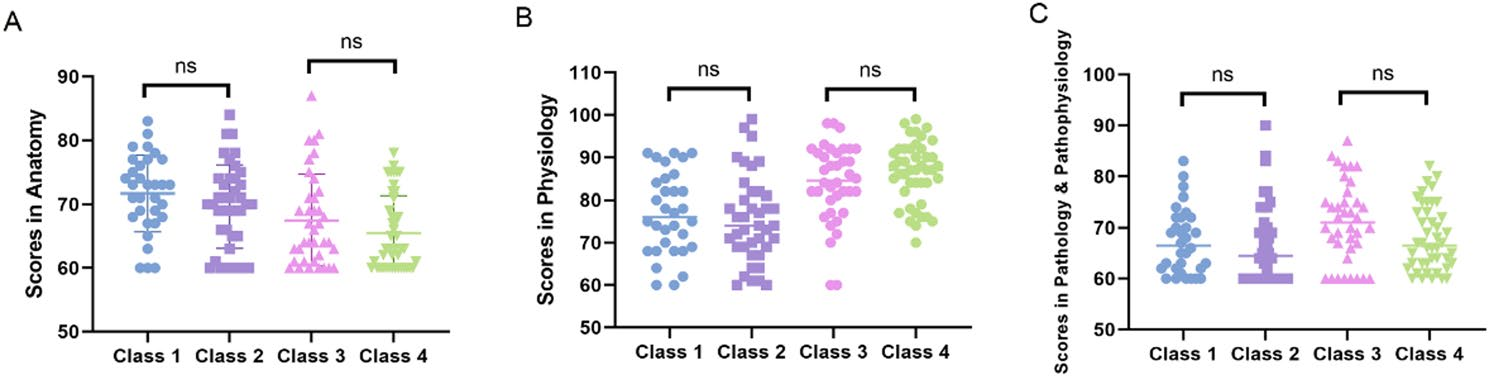
Academic performance in prerequisite courses for *General Surgery*. **A** *Anatomy*. **B** *Physiology*. **C** *Pathology & Pathophysiology*

**Fig. 3:**
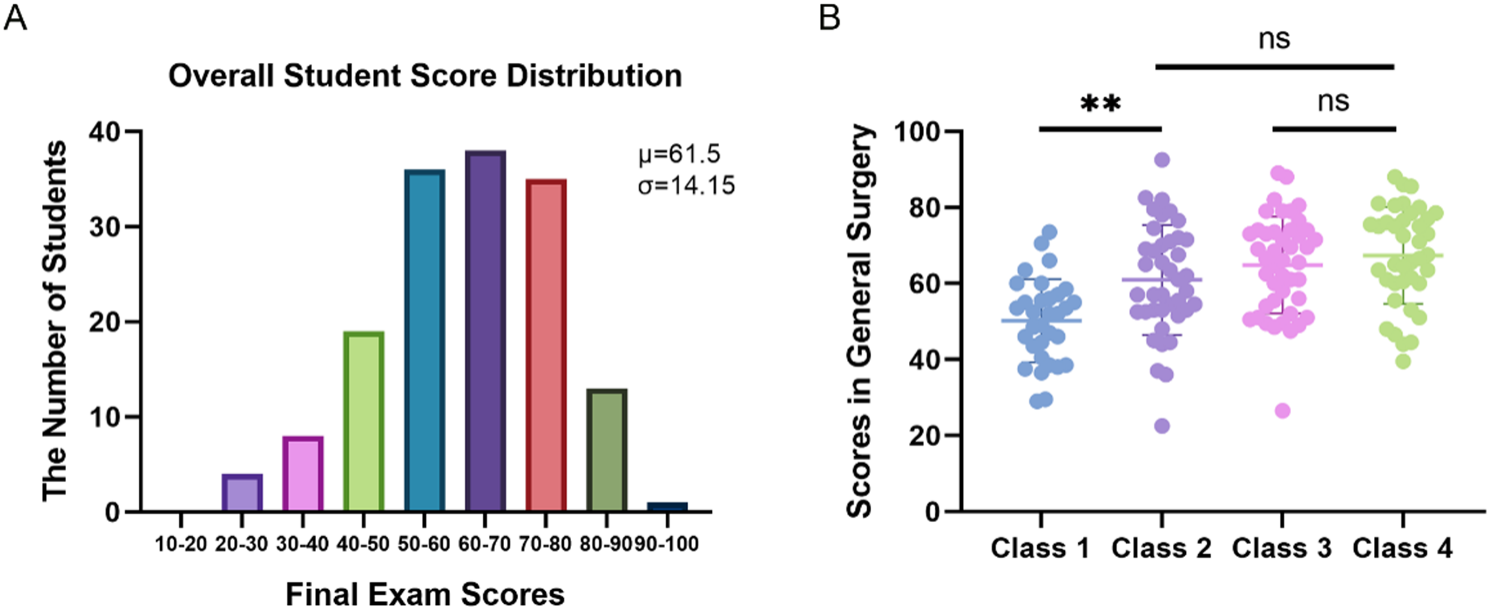
Examination Results of Students. **A** Number of Students in Different Score Ranges for the Final Exam. The average score was 61.5 points, with a standard deviation of 14.15. **B** Final examination scores in *General Surgery*. *p* < 0.05 indicates a significant difference. **p < 0.01

This quasi-experimental investigation employed pre-existing class cohorts to examine instructional efficacy in surgical education. To mitigate potential baseline disparities inherent in non-randomized grouping, we conducted comparative analysis of students’ prior academic performance across three foundational courses (*Anatomy*,* Physiology*,* and Pathology & Pathophysiology*). Initial assessment revealed statistically comparable achievement between Class 1 and Class 2 (*p* = 0.47), with analogous equivalence observed between Class 3 and Class 4 (*p* = 0.39) (Fig. 2**)**, confirming uniform preparatory knowledge bases for the subsequent *General Surgery* curriculum. Following discipline-specific instruction under differentiated pedagogical approaches, all student cohorts completed standardized final examinations. The resultant score distribution spanned from 22.5 to 92.5 (mean = 61.5 ± 14.15), exhibiting concentration of approximately 68% of scores within the 50–80 range (Fig. 3A), indicative of predominant intermediate-level competency with moderate dispersion. The average exam scores for Classes 1, 2, 3, and 4 were 51.5 ± 10.3, 61.6 ± 13.8, 65.1 ± 12.1, and 67.7 ± 12.7, respectively. Notably, while both Class 1 and Class 2 received clinician-taught instruction, the implementation of personalized tutoring in Class 2 yielded significant performance enhancement relative to Class 1 (*p* < 0.01). This intervention brought the Class 2 cohort to statistical parity with faculty-taught Classes 3 and 4 (*p* > 0.05, Fig. 3B), confirming that personalized tutoring support compensates for instructional gaps in clinician-taught courses.

### Impact of teaching modalities on final exam scores

Based on the final exam scores in *General Surgery*, students were categorized as “Good” (top 30%) or “Fail” (bottom 20%). Subsequent binary logistic regression compared the “Fail” versus “Non-Fail” groups (Table 1). Univariate analysis demonstrated that Class 1 had significantly reduced odds of academic success (OR = 0.240; 95% CI: 0.082–0.702; *p* = 0.009) relative to Class 2, indicating substantially lower probability of achieving passing grades. In contrast, Class 3 and Class 4 showed non-significant odds ratios of 1.343 (95% CI: 0.410–4.398) and 1.400 (95% CI: 0.403–4.862) respectively (*p* > 0.05), establishing statistical equivalence among Classes 2, 3, and 4. This pattern was replicated in analyses of “Good” performance attainment (Table 2).

**Table 1:** Univariate logistic regression analysis of failing and unfailing students

Characteristics	Total(*N*)	Univariate analysis	
		Odds Ratio (95% CI)	*P* value
Teaching mode	154		
Clinician-Taught and Personalized Tutoring (Class 2)	40	Reference	
Clinician-Taught (Class 1)	32	0.240 (0.082–0.702)	0.009
Faculty-Taught and Personalized Tutoring (Class 4)	38	1.400 (0.403–4.862)	0.596
Faculty-Taught (Class 3)	44	1.343 (0.410–4.398)	0.626

The analysis was performed using R version 4.2.1. The rms package (version 6.4.0) was used for model fitting, and the Resource Selection package (version 0.3–5.3) was used for goodness-of-fit testing

**Table 2:** Univariate logistic regression analysis of good and non-good students

Characteristics	Total(*N*)	Univariate analysis	
		Odds Ratio (95% CI)	*P* value
Teaching mode	154		
Clinician-Taught and Personalized Tutoring (Class 2)	40	Reference	
Clinician-Taught (Class 1)	32	0.156 (0.032–0.758)	0.021
Faculty-Taught and Personalized Tutoring (Class 4)	38	2.333 (0.922–5.904)	0.074
Faculty-Taught (Class 3)	44	1.469 (0.592–3.645)	0.407

The analysis was performed using R version 4.2.1. The rms package (version 6.4.0) was used for model fitting, and the Resource Selection package (version 0.3–5.3) was used for goodness-of-fit testing

### Differences in teaching characteristics between different types of instructors

**Fig. 4:**
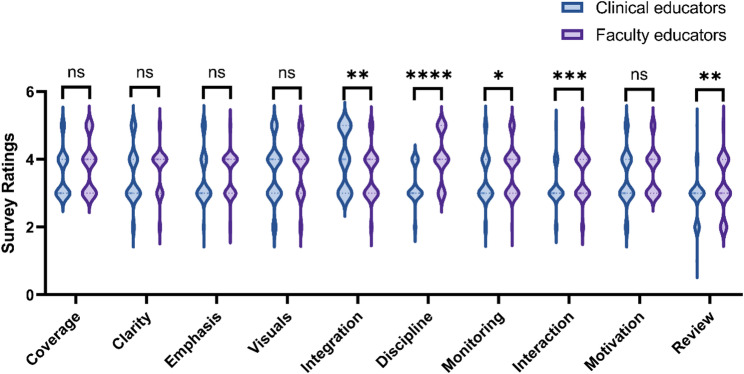
Contrasting teaching profiles of clinical educators and faculty educators. *p* < 0.05 indicates a significant difference. *p<0.05, **p < 0.01, ***p < 0.001,****p < 0.0001

A comparative analysis of teaching characteristics between clinical educators and faculty educators was conducted through questionnaire surveys (Table 3). The results revealed significant differences in teaching approaches between the two groups. Clinical educators demonstrated significantly better performance in “integrating theory with clinical practice” (*p* < 0.05), while showing significantly weaker performance in four dimensions compared to faculty educators (all *p* < 0.05): rigor in classroom discipline, monitoring of student comprehension, frequency of teacher-student interactions, and systematic review of prior knowledge (Fig. 4). This distinct pattern suggests that faculty educators place greater emphasis on pedagogical process management and systematic knowledge acquisition, including maintaining classroom discipline, closely tracking learning progress, enhancing teacher-student interactions, and regularly reviewing teaching content. In contrast, the strength of clinical educators lies primarily in their depth of integrating theoretical knowledge with clinical practice.

**Table 3:** Comparison of teaching evaluation scores between clinical educators and faculty educators

Dimensions	Clinical educators		Faculty educators		*P* value
	Mean	SD	Mean	SD	
Coverage	3.58	0.69	3.78	0.75	0.108
Clarity	3.54	0.82	3.68	0.65	0.088
Emphasis	3.46	0.75	3.57	0.61	0.349
Visuals	3.60	0.85	3.76	0.78	0.185
Integration	3.96	0.86	3.57	0.72	0.008
Discipline	3.24	0.54	4.06	0.73	<0.0001
Monitoring	3.40	0.73	3.70	0.71	0.011
Interaction	3.17	0.58	3.49	0.67	0.001
Motivation	3.44	0.79	3.66	0.69	0.091
Review	2.88	0.63	3.18	0.77	0.007

Scores were based on a 5-point Likert scale (1 = Strongly Disagree to 5 = Strongly Agree)

*SD* Standard deviation

## Discussion

The significantly lower academic performance observed in Class 1 (Clinician-taught) compared to other classes warrants careful interpretation. This disparity likely stems from inherent differences in teaching approaches between clinical educators and faculty educators. Clinical educators undeniably possess profound content knowledge and excel at clinical integration, as confirmed by our data. However, effective teaching requires translating this expertise into pedagogically sound practices. Our questionnaire data reveal that clinical educators showed relative weaknesses in foundational pedagogical domains, specifically in maintaining classroom discipline, monitoring student comprehension, facilitating teacher-student interactions, and systematically reviewing prior knowledge. These quantified findings clarify the core issue: clinical educators undeniably possess profound content knowledge, but effective teaching requires the translation of this expertise into pedagogically sound practices. The identified weaknesses are not minor adjuncts but are central to the process of knowledge transfer. For vocational medical students, who often enter with heterogeneous and less robust academic foundations, the structured, disciplined, and interactive learning environment—which the data show is more consistently provided by faculty educators—is not merely beneficial but essential for building conceptual understanding.

The rotating model of multiple clinical educators, as opposed to a single dedicated faculty educator, likely intensified these challenges by disrupting instructional continuity and the formation of stable teacher-student relationships, which are crucial for identifying and addressing learning gaps over time. The dramatic remediation achieved in Class 2 (Clinician-taught with Personalized Tutoring) powerfully supports this interpretation. The tutor did not provide new clinical information but rather supplied the missing pedagogical scaffolding—the consistent oversight and interactive support—that the rotating clinical educators could not consistently deliver, thereby unlocking the students’ ability to benefit from the clinical educators’ expert knowledge.

Conversely, the absence of a tutoring benefit in the faculty-taught classes suggests that faculty educators, through their training and experience, have already integrated effective pedagogical practices into their standard teaching. They likely possess a more developed PCK, enabling them to anticipate student difficulties, structure content for optimal uptake, and conduct formative assessments seamlessly within the regular curriculum, making supplemental tutoring redundant.

However, these results should not be interpreted as inherent deficiencies in the teaching abilities of clinician educators. Rather, the disparity in academic outcomes appears to reflect a critical mismatch between the default teaching style of clinical educators and the distinct learning needs of the vocational student population. These students typically enter the program with less robust foundational knowledge (as reflected in lower college entrance exam scores) and face a compressed, high-intensity curriculum. In this context, the highly structured, disciplined, and consistently monitored learning environment provided by faculty educators may simply represent a more suitable pedagogical approach for ensuring knowledge acquisition. This should not overshadow the indispensable value of clinical educators’ strengths in clinical integration, which remains a cornerstone of medical education.

The optimal solution may lie in hybrid models that combine clinical educators’ practical expertise with structured educational support, particularly for foundational courses where conceptual mastery is critical. Future models could involve co-teaching, targeted faculty development for clinical educators in core pedagogical skills, or integrating clinical educators into a faculty-designed curriculum to ensure students benefit from both deep clinical insight and effective knowledge acquisition.

## Limitations

This study has several limitations that need to be acknowledged. First, due to constraints in the actual teaching arrangements, we were unable to use a randomized grouping method and instead based the study on the existing class divisions. Although the analysis of students’ performance in prerequisite courses such as *Anatomy*, *Physiology*, and *Pathology & Pathophysiology* confirmed that the foundational knowledge levels of students across the classes were comparable (*p* > 0.05), this quasi-experimental design may not fully control for potential confounding factors.

Second, this study used the written exam in *General Surgery* as the primary evaluation metric. While this assessment method effectively measures students’ mastery of basic theoretical knowledge, it does not fully capture clinical practice abilities, a key dimension of medical education. Consequently, the clinical reasoning skills demonstrated by clinical educators during their teaching may not have been adequately reflected [17]. Future research should incorporate practical assessment methods, such as Objective Structured Clinical Examinations (OSCEs), to more comprehensively evaluate the effectiveness of different teaching models.

Moreover, the follow-up period in this study was limited to one semester, and such a short-term evaluation may not fully reflect the actual value of different teaching methods. Finally, all the participating classes were from the same institution. While this helps control for institutional-level confounding factors, it also somewhat limits the generalizability of the study’s findings.

## Conclusion

This study demonstrates that personalized tutoring effectively improves written exam performance in clinician-taught classes to levels comparable with faculty-taught classes, while faculty-taught classes show no significant benefit from additional tutoring. These differences primarily stem from distinct teaching characteristics: faculty educators excel in systematic knowledge delivery whereas clinical educators emphasize clinical practice integration. We recommend targeted pedagogical training for clinical educators and implementation of a clinician-faculty collaborative teaching model to better serve vocational medical students’ learning needs.

## Supplementary Information

Supplementary Material 1.

## Data Availability

These data during the current study are not publicly available due to confidentiality but are available from the corresponding author on reasonable request.
