# Supplementary material for: Personalized tutoring narrows the clinician-faculty gap in General Surgery exam scores among vocational medical students
Source: BMC Med Educ. 2025 Dec 1;26:10. doi: 10.1186/s12909-025-08371-5 (PMC12771876; doi:10.1186/s12909-025-08371-5)
Supplement: Supplementary file 1 — Supplementary Material 1. [file 12909_2025_8371_MOESM1_ESM.docx]

**Survey Questionnaire**

**Questionnaire Instructions**

This questionnaire aims to collect and analyze students' evaluations of teachers' classroom instruction as well as students' self-assessments of their usual classroom performance. Through this data, we hope to gain a deeper understanding of students' perspectives on classroom teaching, thereby providing valuable insights for improving teaching methods.

Your participation in this study is entirely voluntary. You may choose to decline participation or withdraw at any stage of completing the questionnaire without any negative consequences. All collected data will be kept strictly confidential and used solely for the analysis in this study.

There are no known risks associated with this study, and participation will not cause you any physical discomfort or risk.

**Basic Information**

Your student ID:

Your class:

A. Class 1

B. Class 2

C. Class 3

D. Class 4

**For the following questions, please rate them on a scale of 1 to 5, where:**

1 = "Strongly Disagree"

2 = "Disagree"

3 = "Neutral"

4 = "Agree"

5 = "Strongly Agree"

**1. Do you think the teacher's lectures covered the key points for the final exam this semester?**

A. 1 (Strongly Disagree)

B. 2 (Disagree)

C. 3 (Neutral)

D. 4 (Agree)

E. 5 (Strongly Agree)

**2. How clear was the teacher's explanation of the course material during class?**

A. 1 (Very Unclear)

B. 2 (Unclear)

C. 3 (Neutral)

D. 4 (Clear)

E. 5 (Very Clear)

**3.How well did the teacher emphasize key and difficult concepts during lectures?**

A. 1 (Not at all)

B. 2 (Poorly)

C. 3 (Neutral)

D. 4 (Well)

E. 5 (Very Well)

**4. How engaging and visually effective were the teaching materials (e.g., slides) used in class?**

A. 1 (Not at all)

B. 2 (Slightly)

C. 3 (Neutral)

D. 4 (Engaging)

E. 5 (Very Engaging)

**5. To what extent did the teacher integrate theoretical knowledge with clinical practice?**

A. 1 (Not at all)

B. 2 (Minimally)

C. 3 (Neutral)

D. 4 (Well-integrated)

E. 5 (Very Well-integrated)

**6. How strict was the teacher in maintaining classroom discipline?**

A. 1 (Very Lenient)

B. 2 (Lenient)

C. 3 (Neutral)

D. 4 (Strict)

E. 5 (Very Strict)

**7. Did the teacher pay attention to whether students understood the material?**

A. 1 (Not at all)

B. 2 (Rarely)

C. 3 (Sometimes)

D. 4 (Often)

E. 5 (Always)

**8. How was the teacher's interaction with students during class?**

A. 1 (Very Poor)

B. 2 (Poor)

C. 3 (Neutral)

D. 4 (Good)

E. 5 (Excellent)

**9. Did the teacher make efforts to enhance students' motivation to learn?**

A. 1 (Not at all)

B. 2 (Minimally)

C. 3 (Occasionally)

D. 4 (Often)

E. 5 (Always)

**10. Did the teacher review previous knowledge when necessary?**

A. 1 (Never)

B. 2 (Rarely)

C. 3 (Sometimes)

D. 4 (Often)

E. 5 (Always)

**The following table is not visible to students**

**Keyword Reference Table for the 10 Questions**

| **Question No.** | **Question Summary** | **Keywords** | **Explanation** | **Analysis Purpose** |
| --- | --- | --- | --- | --- |
| Q1 | Course content coverage of exam points | **Coverage** | Alignment between teaching content and exams | Verify consistency between teaching and assessment |
| Q2 | Clarity of knowledge point explanations | **Clarity** | How well the teacher explains concepts | Evaluate basic teaching quality |
| Q3 | Emphasis on key/difficult points | **Emphasis** | Strength of focus on core content | Identify effectiveness of teaching strategies |
| Q4 | Engagement level of teaching materials | **Visuals** | Appeal of instructional media | Analyze the impact of teaching aids on learning |
| Q5 | Integration of theory with clinical practice | **Integration** | Closeness of theory-practice connection | Distinguish teaching styles between different instructors |
| Q6 | Strictness of classroom discipline | **Discipline** | Rigor of classroom management | Explore the relationship between discipline and performance |
| Q7 | Attention to student comprehension | **Monitoring** | Teacher's tracking of learning outcomes | Assess implementation of formative evaluation |
| Q8 | Teacher-student interaction | **Interaction** | Frequency of participation and feedback | Compare differences in interactive teaching methods |
| Q9 | Efforts to boost learning motivation | **Motivation** | Teacher's attempts to engage students | Analyze drivers of student participation |
| Q10 | Necessity of reviewing past knowledge | **Review** | Maintenance of knowledge continuity | Verify the role of review strategies |
